# Global species delimitation of the cosmopolitan marine littoral earthworm *Pontodrilus litoralis* (Grube, 1855)

**DOI:** 10.1038/s41598-024-52252-8

**Published:** 2024-01-19

**Authors:** Teerapong Seesamut, Yuichi Oba, Parin Jirapatrasilp, Svante Martinsson, Maria Lindström, Christer Erséus, Somsak Panha

**Affiliations:** 1 Department of Biology, Faculty of Science, Rangsit University, Pathumthani, 12000 Thailand; 2 Department of Environmental Biology, Chubu University, Kasugai, 487-8501 Japan; 3 Animal Systematics Research Unit, Department of Biology, Faculty of Science, Chulalongkorn University, Bangkok, 10330 Thailand; 4 Department of Biological and Environmental Sciences, University of Gothenburg, Box 463, 405 30 Göteborg, Sweden; 5 Academy of Science, The Royal Society of Thailand, Bangkok, 10300 Thailand

**Keywords:** Evolution, Molecular biology, Zoology

## Abstract

The marine littoral earthworm *Pontodrilus litoralis* (Grube, 1855) is widely distributed and is reported as a single species. This study utilized an integrative taxonomic approach based upon morphological examination, phylogenetic reconstruction, and molecular species delimitation, to test whether the taxon is a single species or a species complex. For this, a total of 114 *P. litoralis* specimens collected from North America, Africa, Australia and Oceania, Europe and Asia were used. The phylogenetic analyses revealed deeply divergent mitochondrial lineages and a high level of genetic diversity among *P. litoralis* populations. Both single and multi-locus species delimitation analyses yielded several molecular operational taxonomic units. Therefore, due to the homogeneity of morphological characteristics, it is likely that the morphospecies *P. litoralis* is a complex of four or more cryptic species, suggesting that more sampling is required and that the population structure genetic data and gene flow need to be investigated.

## Introduction

The bioluminescent earthworm *Pontodrilus litoralis* (Grube, 1855) has been reported as a cosmopolitan species, inhabiting marine littoral ecosystems in the sub-temperate and tropical coastal areas of the Atlantic, Pacific, and Indian oceans^1–4^, and is reported to be both arenicolous and limicolous. The first description of this littoral earthworm, named as *Lumbricus litoralis* by Grube (1855)^5^, was based on the morphological characteristics of a Mediterranean sample from the Villefranche-sur-Mer (formerly Villafranca) on Côte d’Azur, France. The genus *Pontodrilus* was first established by Perrier (1874)^6^ who also described *P. marionis* Perrier, 1874; however, Beddard (1895)^7^ subsequently synonymized *P. marionis* with *L. litoralis*. Easton (1984)^1^ then provided an extensive list of *P. litoralis* synonyms and references to the taxonomic literature and concluded that *P. litoralis* is a single species which is highly variable. Although, a few other morphologically distinct species of *Pontodrilus* have been discovered, only two species of *Pontodrilus*, including *P. litoralis*, have been reported from Thailand and peninsular Malaysia^2^. Chen et al. (2021)^8^ hypothesized that the widespread populations of *P. litoralis* throughout the world resulted from their transport by currents, which is congruent with Blakemore’s (2007)^9^ suggestion that the wide distribution of *P. litoralis* is due to the transport of ships’ sand-ballast, and the natural rafting of euryhaline cocoons. The wide range of salinity tolerance of *P. litoralis*, shown experimentally by Seesamut et al., (2022)^10^, may have facilitated this species’ wide distribution pattern.

Molecular (DNA) taxonomy in earthworms has mostly used a single marker gene, in particular the mitochondrial cytochrome *c* oxidase subunit 1 (*COI*) gene. When such a marker is used to identify species, the method is referred to as DNA barcoding^11–15^. However, many earthworm studies have implemented both nuclear and mitochondrial genes in phylogenetic species delimitation^16–19^. Widely used methods based on single-locus sequences are, e.g., Automatic Barcode Gap Discovery (ABGD)^20^, Assemble Species by Automatic Partitioning (ASAP)^21^, Bayesian implementation of Poisson Tree Processes model (bPTP)^22^ and General Mixed Yule Coalescent model (GMYC)^23^; for more details, see the review by Martinsson & Erséus (2021)^24^ and Goulpeau et al., (2022)^25^. However, for sexually reproducing species, multiple-locus delimitation, which takes the evolution of more than one gene into account, may be more reliable for testing hypotheses of speciation events; for instance, congruent nodes in the comparison between one nuclear and one mitochondrial gene tree are more supportive of a speciation event (ceased gene flow) than are incongruent nodes, which are evidence of gene flow between individuals belonging to different “mitochondrial” (= maternal) lineages^19,26,27^.

Despite the worldwide distribution records of *P. litoralis*, scientists still believe that it is a single species, and this is largely based on morphological characteristics. Variation in the body size between populations in Asia has been studied, but these marked difference in the morphometrics of *P. litoralis* across geographic populations did not correlate with their genetic differences (*COI*). Rather, it was suggested that *P. litoralis* is a single species^3^. In this study, we aimed to test the hypothesis that the worldwide distributed earthworm *P. litoralis* is a single species as proposed by Easton (1984) and Seesamut (2019)^1,3^. The earthworms were collected from North America, Australia and Oceania, Europe, Africa, and Asia (East and Southeast Asia), and morphological examination, phylogenetic analysis, and species delimitation using the methods mentioned above plus multi-locus delineation using Bayesian phylogenetics and phylogeography^28,29^ were conducted.

## Results

**Figure 1:**
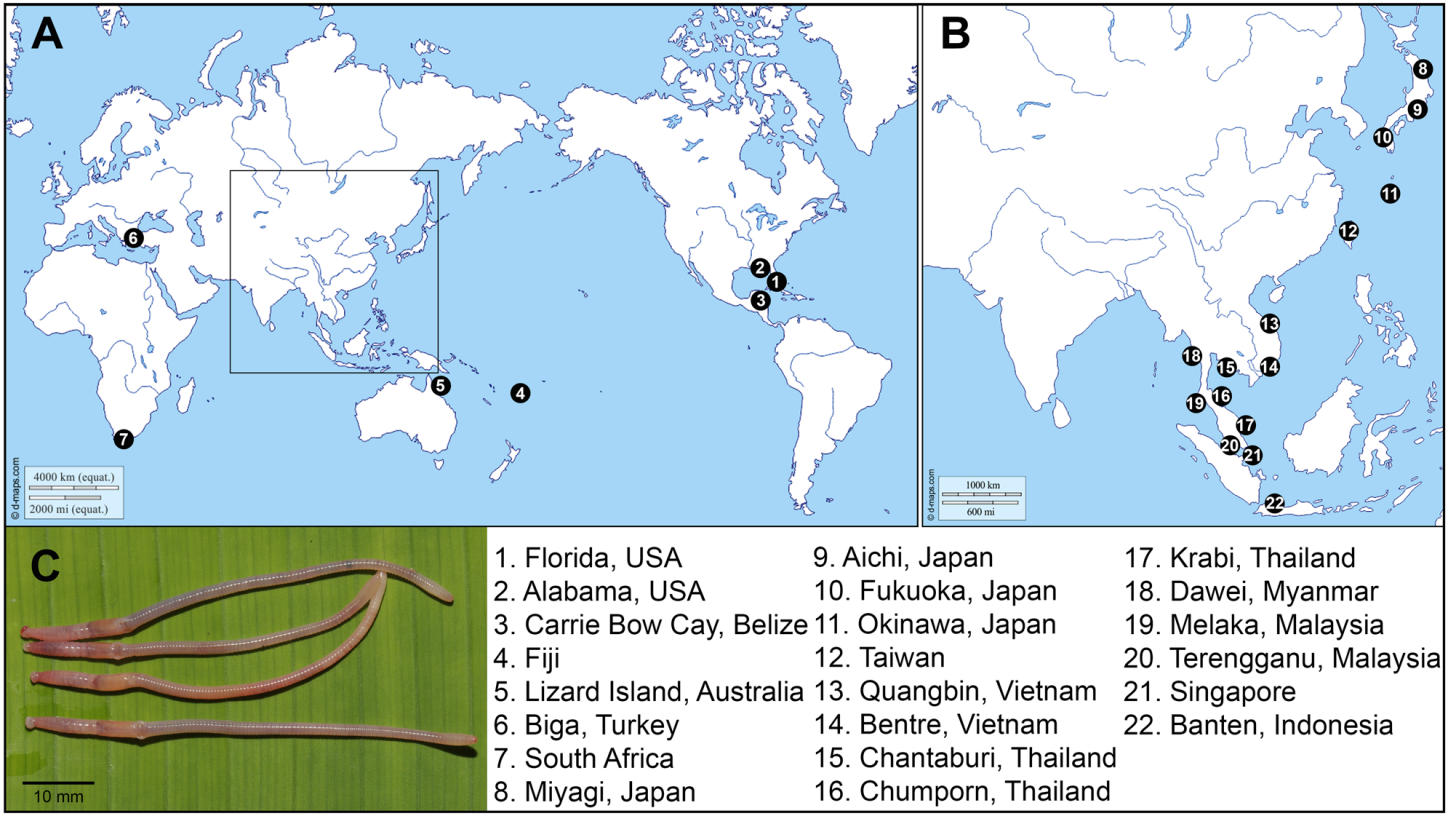
(**A** and **B**) Map showing the sampling sites of *P. litoralis*. The map is based on a map from D-maps (available at https://d-maps.com/carte.php?num_car=3228&lang=en), map was edited in Adobe Photoshop. (**C**) Photograph of *P. litoralis* from Thailand (photograph by Teerapong Seesamut).

**Figure 2:**
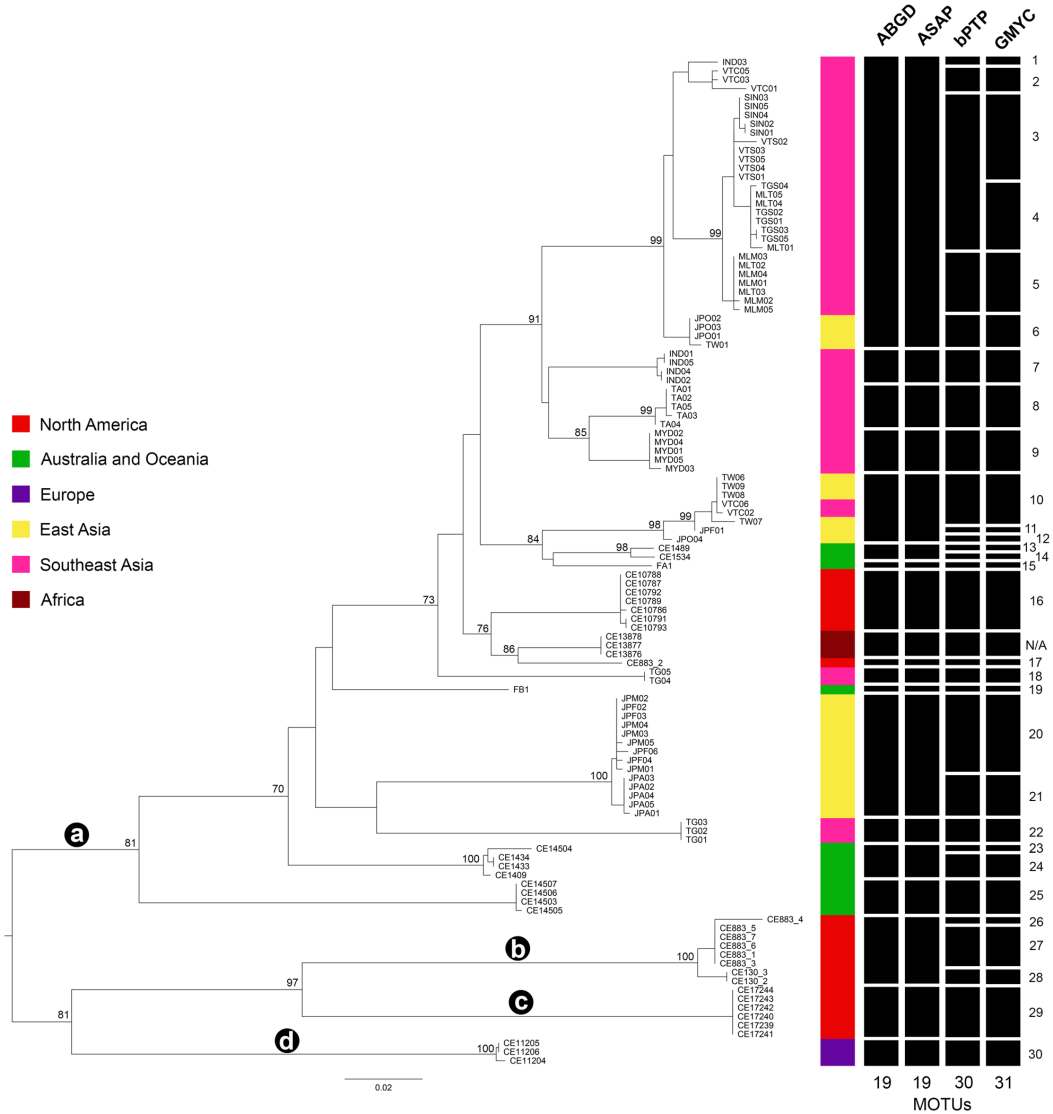
A ML phylogenetic tree of *P. litoralis* based on the *COI* fragment sequence (658 bp) and the species delimitation clustering results. The nodes with ML bootstraps > 70% are considered well-supported. The scale bar indicates the branch length. ABGD, automated barcode gap discovery; ASAP, Assemble Species by Automatic Partitioning; bPTP, Bayesian implementation of Poisson Tree Processes model; GMYC, generalized mixed Yule coalescent model. The numbering is the input MOTUs of the BPP analyses, the letters a—d are the four most conservative MOTUs suggested by BPP.

We obtained a total of 114 *COI* sequences of *P. litoralis* which included 22 specimens from North America, three from Africa, 12 from Australia and Oceania, three from Europe, and 74 from Asia (24 from East Asia and 50 from Southeast Asia) (Fig. 1, Table 1). The final aligned dataset, comprised of 658 bp sequence fragments, contained a total of 392 invariable (monomorphic) sites, 210 variable (polymorphic) sites (total number of mutations is 283), and 119 parsimony informative sites. The result yielded a total of 52 haplotypes, with a haplotype (gene) diversity of 0.978 and a nucleotide diversity (Pi) of 0.09838. All sequences are deposited in GenBank (Table 1). Based on *P. litoralis* samples from different geographic distributions, the *COI* phylogenetic tree revealed a high genetic diversity, and the *COI-*based species delimitations revealed that the 114 specimens were divided into 19 MOTUs by ABGD and ASAP, whereas the bPTP and GMYC methods yielded 30 and 31 MOTUs, respectively (Fig. 2).

**Table 1:** List of *P. litoralis* specimens examined in this study, and accession numbers of the *COI* and ITS2 sequences. * Juvenile stage; ** Only tail was collected.

Regions	Collection locality	Abbr	*COI*	ITS2
North America	Craig Key, Florida, USA	CE130_2*	OR889174	OR897723
		CE130_3*	OR889175	-
	Fort Pierce, Florida, USA	CE883_1*	OR889195	OR897734
		CE883_2*	OR889196	OR897735
		CE883_3*	OR889197	OR897736
		CE883_4*	OR936652	OR897737
		CE883_5*	OR936650	OR897738
		CE883_6*	OR889198	OR897739
		CE883_7*	OR936651	–
	Cedar Pt, Alabama, USA	CE10786	OR889163	OR897715
		CE10787	OR889164	OR897716
		CE10788	OR889165	OR897717
		CE10789	OR889166	OR897718
		CE10791	OR889167	OR897719
		CE10792	OR889168	OR897720
		CE10793	OR889169	OR897721
	Carrie Bow Cay, Belize	CE17239	OR889189	OR897731
		CE17240	OR889190	OR897732
		CE17241	OR889191	OR897733
		CE17242*	OR889192	-
		CE17243*	OR889193	-
		CE17244*	OR889194	-
South Africa	South Africa	CE13876	OR889176	-
		CE13877	OR889177	-
		CE13878	OR889178	-
Australia and Oceania	Lizard Is., Australia	CE1409*	OR889179	OR897724
		CE1433*	OR889180	OR897725
		CE1434*	OR889181	-
		CE1489*	OR889187	OR897729
		CE1534*	OR889188	OR897730
		CE14503	OR889182	OR897726
		CE14504	OR889183	OR897727
		CE14505	OR889184	-
		CE14506	OR889185	OR897728
		CE14507	OR889186	-
	Fiji	FA1	OR889162	OR897740
		FB1	OR889173	OR897741
Europe	Biga, Turkey	CE11204	OR889170	OR897722
		CE11205	OR889171	-
		CE11206**	OR889172	-
Southeast Asia	Chantaburi, Thailand	TG01	OR889248	OR897791
		TG02	OR889249	OR897792
		TG03	OR889250	OR897793
		TG04	OR889251	OR897794
		TG05	OR889252	-
	Krabi, Thailand	TA01_A TA01_B	OR889243	OR897787 OR897788
		TA02	OR889244	OR897789
		TA03	OR889245	-
		TA04	OR889246	OR897790
		TA05	OR889247	-
	Chumporn, Thailand	TGS01	OR889253	OR897795
		TGS02	OR889254	-
		TGS03_A TGS03_B	OR889255	OR897796 OR897797
		TGS04_A TGS04_B	OR889256	OR897798 OR897799
		TGS05	OR889257	-
	Dawei, Myanmar	MYD01	OR889233	OR897775
		MYD02	OR889234	OR897776
		MYD03	OR889235	OR897777
		MYD04	OR889236	OR897778
		MYD05	OR889237	–
	Singapore	SIN01_A SIN01_B	OR889238	OR897779 OR897780
		SIN02_A SIN02_B	OR889239	OR897781 OR897782
		SIN03_A SIN03_B	OR889240	OR897783 OR897784
		SIN04_A SIN04_B	OR889241	OR897785 OR897786
		SIN05	OR889242	–
	Bentre, Indonesia	IND01	OR889199	OR897742
		IND02	OR889200	OR897743
		IND03	OR889201	OR897744
		IND04	OR889202	OR897745
		IND05	OR889203	-
	Melaka, Malaysia	MLM01_A MLM01_B	OR889223	OR897765 OR897766
		MLM02_A MLM02_B	OR889224	OR897767 OR897766
		MLM03	OR889225	OR897769
		MLM04_A MLM04_B	OR889226	OR897770 OR897771
		MLM05	OR889227	–
	Terengganu, Malaysia	MLT01	OR889228	OR897772
		MLT02	OR889229	–
		MLT03	OR889230	OR897773
		MLT04	OR889231	OR897774
		MLT05	OR889232	–
	Quangbinh, Vietnam	VTC01	OR936654	OR897806
		VTC02	OR889262	OR897807
		VTC03	OR889263	OR897808
		VTC05	OR889264	OR897809
		VTC06	OR889265	–
	Bentre, Vietnam	VTS01	OR889266	OR897810
		VTS02	OR936655	OR897811
		VTS03	OR889267	–
		VTS04	OR889268	OR897812
		VTS05	OR889269	–
East Asia	Miyagi, Japan	JPM01	OR889214	OR897756
		JPM02	OR889215	OR897757
		JPM03	OR889216	OR897758
		JPM04	OR889217	OR897759
		JPM05	OR889218	-
	Okinawa, Japan	JPO01	OR889219	OR897760
		JPO02	OR889220	OR897761
		JPO03	OR889221	OR897762
		JPO04_A JPO04_B	OR889222	OR897763 OR897764
	Aichi, Japan	JPA01	OR889204	OR897746
		JPA02	OR889205	OR897747
		JPA03	OR889206	OR897748
		JPA04	OR889207	OR897749
		JPA05	OR889208	OR897750
	Fukuoka, Japan	JPF01	OR889209	OR897751
		JPF02	OR889210	OR897752
		JPF03	OR889211	OR897753
		JPF04	OR889212	OR897754
		JPF06	OR889213	OR897755
	Taiwan	TW01	OR936653	OR897800
		TW06	OR889258	OR897801
		TW07	OR889259	OR897802
		TW08_A TW08_B	OR889260	OR897803 OR897804
		TW09	OR889261	OR897805

**Figure 3:**
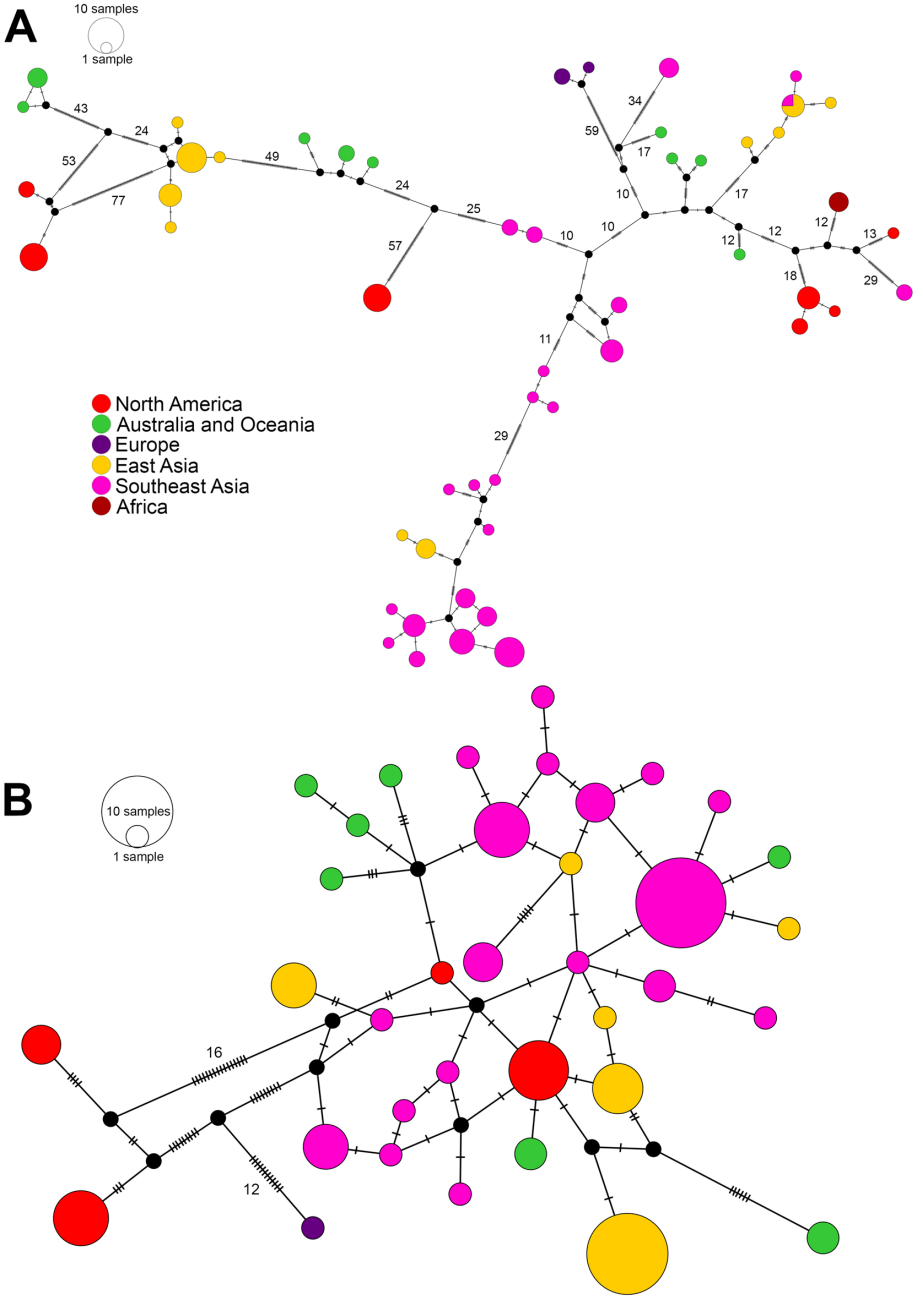
Haplotype networks for (**A**) *COI* sequences (658 bp) and (**B**) ITS2 sequences (437 bp) of *P. litoralis*. Lines with dashes and numbers between circles represent the number of mutational steps between two haplotypes. The number of samples in each haplotype corresponds to the size of the circles in the legend.

The *COI* marker showed a higher variability than the ITS2. The *COI* haplotype network shows that 52 haplotypes were detected in 114 individuals, with each (location) population having its own single haplotype. Only one haplotype was shared across two locations from different countries: Quangbinh (Vietnam) and Taiwan (Fig. 3A). The ITS2 haplotype network showed a total of 36 haplotypes from 98 individuals (Fig. 3B). The highest numbers of mutational steps are 77 and 16 in *COI* and ITS2, respectively.

**Figure 4:**
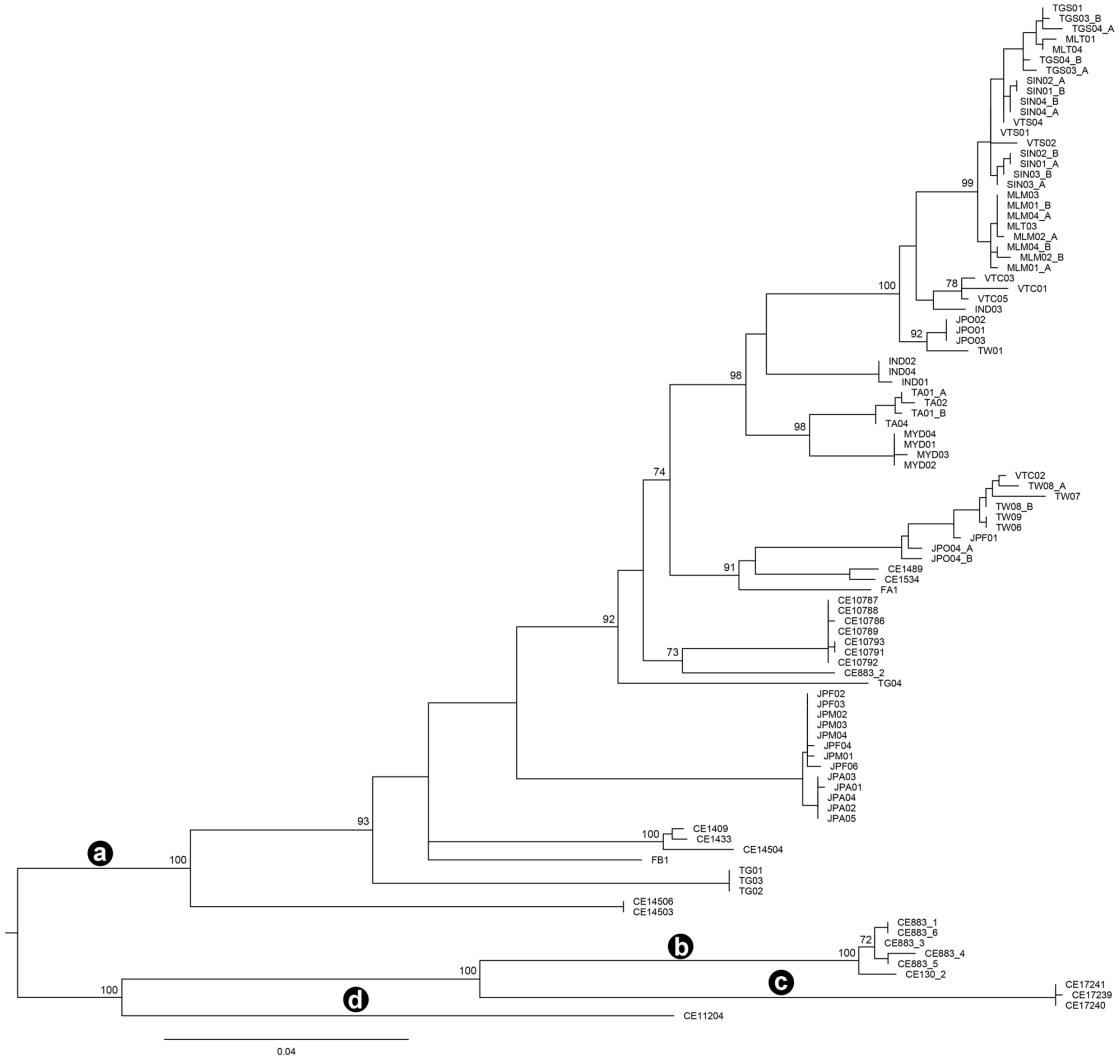
A ML concatenated tree of *COI* and ITS2. Nodes with ML bootstraps > 70% are considered well-supported. The scale bar indicates the branch length. The four most conservative MOTUs suggested by the BPP analysis are marked with black circles labelled with a–d respectively.

The phylogenetic relationships observed in the analysis of the concatenated data (*COI* + ITS2) were congruent with the *COI* and ITS2 phylogenetic trees (Figs. 2, 4, and Supplementary Fig. 1). The results of the BPP analyses are summarised in Table 2. In analysis A, B, and C, 17, 3, and 11, respectively, out of the 30 MOTUs are supported with a PP of > 0.95. The only two MOTUs that are supported in all three analyses are MOTU 29 and 30. In one of the three separate analyses of B and C, respectively, maximum support was found for combining a majority of the MOTUs into one. The most conservative estimate would be four MOTUs, i.e., (a) combining MOTUs 1–25, (b) combining MOTUs 26–28, (c) MOTU 29, and (d) MOTU 30 (Figs. 2, 4). There is some support for combining (i) MOTUs 26 and 27 and (ii) MOTUs 26 and 28. Based on these four MOTUs delineated by the BPP, interspecific *COI* uncorrected *p*-distances were calculated, revealing that the genetic divergence among this conservative set of MOTUs ranged from 13.9 to 16.9%.

**Table 2:** List of species delimitation and their posterior probability (PP) given as a mean of three separate runs. The results with > 0.05 PP in at least one analysis are included. Posterior probabilities in bold are considered significant and MOTUs in bold are accepted.

MOTU	A mean PP	B mean PP	C mean PP
**29**	**1.000**	**1.000**	**1.000**
**30**	**1.000**	**1.000**	**1.000**
28	0.788	**0.968**	**0.997**
27	0.768	0.927	**0.988**
22	**1.000**	0.667	**1.000**
25	**1.000**	0.667	**1.000**
20	**0.993**	0.666	**1.000**
21	**0.993**	0.666	**1.000**
26	0.695	0.924	**0.989**
23	0.749	0.638	**0.996**
24	0.749	0.638	**0.996**
18	**1.000**	0.667	0.667
16	**1.000**	0.667	0.667
17	**1.000**	0.667	0.667
19	**1.000**	0.667	0.667
8	**1.000**	0.667	0.667
9	**1.000**	0.667	0.667
7	**0.999**	0.667	0.667
3	**0.998**	0.666	0.667
15	**0.996**	0.667	0.667
4	**0.995**	0.664	0.666
5	**0.985**	0.663	0.666
2	0.719	0.544	0.632
1	0.689	0.527	0.632
13	0.394	0.456	0.615
14	0.394	0.456	0.615
6 10 11 12	0.128	0.506	0.649
13 14	0.605	0.210	0.052
6	0.653	0.100	0.016
12	0.528	0.123	0.035
10	0.586	0.092	0.002
11	0.462	0.065	0.001
1 2	0.278	0.079	0.002
1 2 3 4 5 6 7 8 9 10 11 12 13 14 15 16 17 18 19	0.000	0.000	0.333
**1 2 3 4 5 6 7 8 9 10 11 12 13 14 15 16 17 18 19 20 21 22 23 24 25**	0.000	0.333	0.000
23 24	0.251	0.028	0.004
11 12	0.180	0.032	0.002
26 27	0.137	0.054	0.010
26 28	0.117	0.013	0.001
10 11	0.091	0.010	0.001
6 10	0.078	0.022	0.001
10 11 12	0.043	0.015	0.013
**26 27 28**	0.052	0.008	0.000

Most *P. litoralis* specimens in this study were in the adult stage, and specimens from different collecting localities showed no difference in any distinctive morphological characteristics.

## Discussion

Morphological investigation showed that the external and internal morphology of *P. litoralis* samples in this study correspond to the original description and those recently reported^1,2,5,30^. The analyses of the single-locus phylogeny and mitochondrial species delimitation suggested that *P. litoralis* is a complex of species, which all seem to be cryptic because of the homogeneity in their morphological characteristics. Moreover, a high degree of genetic structuring among different geographical populations of *P. litoralis* is evident. The occurrence of cryptic species in clitellates has frequently been uncovered, which is not surprising as there are few diagnostic morphological features that can be used to distinguish different species^31^. On the other hand, Martinsson et al*.* (2020)^32^ tested the species hypotheses of the enchytraeid worm *Fridericia magna* in Norway and Sweden and concluded that the data for this morphospecies is consistent with it being a single species. This and other examples (below) have shown that high intraspecific mitochondrial genetic distances are also common in clitellates.

In the semi-aquatic freshwater earthworm genus *Glyphidrilus*, ten single and multi-locus species delimitation methods revealed a high degree of incongruence between the genetic structures and morphology-based species identifications^19^. Several publications have examined and reported deeply divergent mitochondrial lineages and a high genetic diversity within well-established earthworm morphospecies^14,17,33–35^. Although the *COI* species delimitation analyses in this study suggested the presence of either 19, 30, or 31 MOTUs within *P. litoralis*, Lohse (2009)^36^ mentioned that geographic population structure is likely to lead to the overestimation of species numbers retrieved from species delimitation analyses. This has also been a critique of the multispecies coalescent methods, such as BPP^37^, and it is possible that this is a reason for our BPP analysis supporting about 20 MOTUs in the majority of runs, but then shifting to supporting much fewer MOTUs in some of the runs. This variation makes the interpretation of the results harder, and we have, therefore, chosen the more conservative estimate of MOTUs. Thus, we suggest that several MOTUs of *P. litoralis* are possibly affected by the bias from those species delimitation methods that analyzed the dataset containing different geographic populations of *P. litoralis*. With respect to the widespread distribution of the littoral earthworm *P. litoralis*, it may be dispersed around the world by humans or naturally be transported by currents^8,9^. Here, we suggest that the cosmopolitan distribution of *P. litoralis* is more likely to be caused by currents as human-mediated dispersal might cause the identical haplotype to be shared across different populations from distant locations^38^, while in our case there is a lack of identical haplotype shared across distant locations (Fig. 3).

For earthworms, we agree that 13% or thereabouts of *COI* interspecific genetic distance between two earthworm MOTUs could be used as a rule-of-thumb threshold to delimit different species^14,19^. Therefore, the most conservative recognition of only four MOTUs retrieved from the BPP analysis would suggest that *P. litoralis* is represented by four different species in our study (lineages a–d in Figs. 2 and 4). However, a much higher number of MOTUs of *P. litoralis* were detected by the different species delimitation methods. There are more than 20 synonyms of *P. litoralis* that have been reported from around the world^1,2^. Thus, in order to assign which synonym belongs to which different clade within the *P. litoralis* species complex, further investigations of type specimens representing all synonyms (or topotypes, in case of old type specimens or those not preserved in ethanol) are needed by implementing DNA taxonomy together with morphological investigation.

In summary, the global scale phylogeny and species delimitation of the cosmopolitan littoral earthworm *P. litoralis* were here investigated by an integrative taxonomic approach, with both single and multi-locus multispecies coalescent-based species delimitation methods. The study revealed several MOTUs within *P. litoralis* based on *COI* species delimitation alone, and this was well supported by the ITS2 data. The phylogenetic tree shows deeply divergent mitochondrial lineages and a high number of haplotypes, especially for *COI*. Without support from morphological characteristics, we suggest that the morphospecies *P. litoralis* is referred to as a cryptic species. Further in-depth studies of the morphology and anatomy of these littoral earthworms, e.g., by using scanning electron microscopy, are required to investigate the potential presence of cryptic morphology, which would provide further evidence for a more precise taxonomic revision of the species complex. Moreover, studies on population genetics and a search for more evidence (or lack) of gene flow and/or reproductive barriers are needed.

## Materials and methods

### Specimen collection and morphological examination

Specimens of *P. litoralis* were collected from several types of habitats, such as sandy beaches, mangrove swamps of the intertidal zone, sanitary sewer links, estuaries, under the trash or leaf litter, and freshwater channels between the mainland and the sea, in Thailand and surrounding countries in Southeast Asia (Fig. 1A,B) since 2007. All specimens were deposited in Chulalongkorn University Museum of Zoology (CUMZ), Thailand. Additional Japanese, Taiwanese, and Fijian specimens deposited in the collection at Chubu University Japan were included in the analyses. These littoral earthworms could be found in sand mixed with seaweed debris in sandy beaches facing the ocean in Taiwan and Japan, ranging from the northernmost record at Matsushima Bay, Miyagi Prefecture, to Aichi Prefecture, Mideast Honshu, Fukuoka Prefecture, Kyushu, and the Ryukyu archipelago. In addition, additional specimens of *P. litoralis* were collected by Christer Erséus and his team from different beaches at Lizard Island (Great Barrier Reef, Australia), Carrie Bow Cay (the barrier reef of the coast of Belize), and from three localities in Southeastern USA: Cedar Point (Alabama), Craig Key (Florida Keys), and Indian River Lagoon at Fort Pierce (Florida), the two latter sites being about 350 km apart. The Australian sites were all in depressions immediately behind the beach sand, while the US and Belizean sites were in the upper intertidal zone on the seaward slope of the beach. Finally, worms were also obtained from Turkey (Biga Peninsula in Marmara Sea; courtesy of Sermin Acik Cinar) and South Africa (Grahamstown; courtesy of Sam James). All specimens were preserved in 80–99% (v/v) ethanol for molecular analyses. For other details of the worms used in the analysis, see Table 1. Morphological identification (Fig. 1C) was made based on taxonomic literature following Easton (1984), Gates (1972), and Seesamut et al., (2018)^1,2,30^. All work with animals was conducted in accordance with the Institutional Animal Care and Use Committee of Khon Kaen University (IACUC-KKU) under approval number IACUC-KKU-32/65.

### DNA extraction, PCR amplification, and DNA sequencing

Voucher specimens of *P. litoralis* from Southeast Asia and Japan, including Taiwan, were used for the extraction of their total genomic DNA from the posterior part of each earthworm using the Lysis Buffer for PCR (Takara) and following the manufacturer's protocol. Two molecular markers were amplified: a fragment of mitochondrial *COI* and the internal transcribed spacer 2 (ITS2) region of the nuclear ribosomal DNA. The *COI* fragment was amplified with the Tks Gflex™ DNA Polymerase (Takara) using universal primers HCO2198 and LCO 1490^39^, while primers 606F (forward) and 1082R (reverse)^40^ were used for ITS2. The PCR mixture was as follows: 1 μL of Tks Gflex DNA Polymerase (1.25 unit/μL), 25 μL of 2 × Gflex PCR buffer (Mg^2+^, dNTP plus), 1 μL each of primers (10 μM), 19.5 μL of sterilized distilled water, and 2.5 μL of crude lysate with Lysis buffer. The PCR thermal cycling was performed as 94 °C for 2 min, followed by 35 amplification cycles of 94 °C for 60 s, 48 °C for 60 s, and 72 °C for 2 min and then followed by a final 72 °C for 5 min. The concentration and quality of the amplicons were examined by 1% (w/v) agarose gel electrophoresis against a DNA standard marker in 1 × TAE buffer and detected under UV transillumination after staining with SYBR® Safe DNA Gel Stain. The samples for which direct sequencing of the nuclear gene markers failed were subjected to subcloning using Promega pGEM-T Easy Vector System (Promega, Cat: A1360) to separate allelic variants before sequencing. The purifying and sequencing of PCR products were done commercially by Macrogen Inc. (Japan).

For the specimens from the remaining localities, DNA was extracted from small pieces of worm tissue with the E.Z.N.A.® Tissue DNA Kit II (Omega Bio-tek), following the instructions for kits requiring OB protease, or in some cases with DNeasy® Blood & Tissue Kit (250) (QIAGEN). For samples extracted with E.Z.N.A., the tubes were incubated at room temperature for five minutes before eluting the DNA. The remaining parts of the specimens were deposited, as vouchers, in the Swedish Museum of Natural History, Stockholm. The extracted DNA was then used to PCR amplify fragments of the *COI* gene and nuclear ITS2 region using puReTaq Ready-To-Go PCR Beads (GE Healthcare). Amplification was done according to the kit instructions. The *COI* sequences were amplified by thermal cycling with an Eppendorf PCR, programmed at 35 cycles of 40 s at 95 °C, 45 s at 45 °C, and 1 min at 72 °C, with an initial denaturation period of 5 min at 95 °C and a final terminal extension period of 8 min at 72 °C. For the ITS region, there were 25 cycles of 30 s at 95 °C, 30 s at 50 °C and 1 min at 72 °C with the same denaturation and extension period as for *COI*. The PCR products were checked with electrophoresis on agarose gel (1%) stained with ethidium bromide (3%), and the successfully amplified PCR products were purified using an E.Z.N.A® Cycle-Pure Kit (GE Healthcare) according to the manufacturer’s instructions, except for 100 μL of CP buffer was used and the final elution was done with 40 μL sterile deionized water. The products were then sent to Macrogen Inc., South Korea, where all samples were sequenced.

### Sequence editing, alignment, phylogenetic reconstruction, and haplotype analysis

To identity and verify the amplified sequences, the obtained sequences were submitted to the BLASTn algorithm to check and compare with other sequences available in the GenBank databases in the National Center for Biotechnology Information- NCBI (https://blast.ncbi.nlm.nih.gov/Blast.cgi). All sequences were reassembled, edited, and aligned in MEGA X^41^ using the MUSCLE algorithm^42^ with default parameters, and then manually checked by eyes.

The phylogenetic analyses of the *COI* gene and the concatenated dataset (*COI* + ITS2) were conducted. The best-fit nucleotide substitution model of each gene fragment for phylogenetic analysis was determined using JModelTest v2.1.10^43^. Phylogenetic trees were reconstructed under maximum likelihood (ML) through the online portal CIPRES Science Gateway^44^ as implemented in RaxML-HPC2 on XSEDE^45^, with 1,000 bootstrapping replicates and default parameter settings. The ML tree based on the RaxML program was constructed under the GTR + CAT model for the best-fit nucleotide substitution. The resulting tree was plotted using FigTree v.1.4.4 (http://tree.bio.ed.ac.uk/software/figtree) and the tree diagram was created in Adobe Photoshop 2020. The ML analysis of the concatenated data (*COI* and ITS*2*) was done after partitioning the concatenated data with Kakusan4^46^. For the haplotype analysis, the NEXUS format was created by DnaSP v.6^47^ and the haplotype networks were constructed in PopArt^48^ using the TCS method^49^. Genetic divergences were examined using uncorrected p-distance as implemented in MEGA X with a bootstrap re-analysis of 1,000 pseudoreplicates.

### Mitochondrial and multi-locus species delimitation analyses

Molecular species delimitation using the *COI* sequences was performed using the ABGD^20^, ASAP^21^, bPTP^22^ and GMYC^23^ methods. The ABGD is a simple method to split a sequence alignment data set into candidate species. We used the ABGD online server with default settings, to divide the specimens into clusters (http://wwwabi.snv.jussieu.fr/public/abgd). The ASAP analysis^21^ was implemented in an online web server (https://bioinfo.mnhn.fr/abi/public/asap/) under Kimura (K80) model. The lowest score was considered^50^. The bPTP analysis was carried out using an online web server (https://species.h-its.org/) with 100,000 MCMC generations. The GMYC method is a likelihood method for delimiting species by fitting within- and between-species branching models to reconstruct gene trees. The initial Bayesian tree was constructed in the BEAST v1.10.4 package^51,52^. All parameter settings were configured in BEAUTi v1.8.4, while Tracer v1.6 was used to check the estimate sample size (ESS) values and run the trace file. Using the ultrametric tree produced by BEAST, the GMYC analysis was performed in the R package splits.

Multi-locus species delimitation was performed using BPP v.3.3^28,29^ on the *COI* and ITS2 datasets used in the ML analysis. The molecular operational taxonomic units (MOTUs) obtained from the GMYC analysis was used as the input as this analysis yielded the highest number of MOTUs, except for one MOTU for which no ITS2 sequence was available and so this MOTU was omitted from the analysis. The joint Bayesian species delimitations and species tree estimations^28,53,54^ were used, and three analyses (A-C) with different population size (estimated by θ) and divergence time (τ0) priors were performed, using the same settings and priors as in Martinsson and Erséus (2018)^55^ and Martinsson et al., (2020)^32^ (A: θ = 2, 400, τ0 = 2, 200; B: θ = 2, 1000, τ0 = 2, 200; C: θ = 2, 2000, τ0 = 2, 200). Each analysis was run for 200,000 generations, discarding the first 4,000 as burn-in, and all analyses were performed three times to confirm consistency between runs. We considered the species delimited with a PP (posterior probability) > 0.95 in all analyses to be well supported.

### Supplementary Information

Supplementary Figure 1.

## Data Availability

Correspondence and requests for materials should be addressed to C.E. or S.P.
