# Supplementary material for: Global species delimitation of the cosmopolitan marine littoral earthworm Pontodrilus litoralis (Grube, 1855)
Source: Sci Rep. 2024 Jan 19;14:1753. doi: 10.1038/s41598-024-52252-8 (PMC10799051; doi:10.1038/s41598-024-52252-8)
Supplement: Supplementary file 1 — Supplementary Figure 1. [file 41598_2024_52252_MOESM1_ESM.pdf]

Supplementary Materials

Global species delimitation of the cosmopolitan marine littoral earthworm *Pontodrilus litoralis* (Grube, 1855)

Teerapong Seesamut[1], Yuichi Oba[2], Parin Jirapatrasilp[3], Svante Martinsson[4], Maria Lindström[4,6], Christer Erséus[4*] and Somsak Panha[3,5*]

[1]*Department of Biology, Faculty of Sciences, Rangsit University, Pathum Thani 12000, THAILAND*
[2]*Department of Environmental Biology, Chubu University, Kasugai 487-8501, JAPAN*
[3]*Animal Systematics Research Unit, Department of Biology, Faculty of Science, Chulalongkorn University, Bangkok 10330, THAILAND*
[4]*Department of Biological & Environmental Sciences, University of Gothenburg, Box 463, SE-405 30, Göteborg, SWEDEN*
[5]*Academy of Science, The Royal Society of Thailand, Bangkok 10300, THAILAND*
[6]*Deceased 2012*

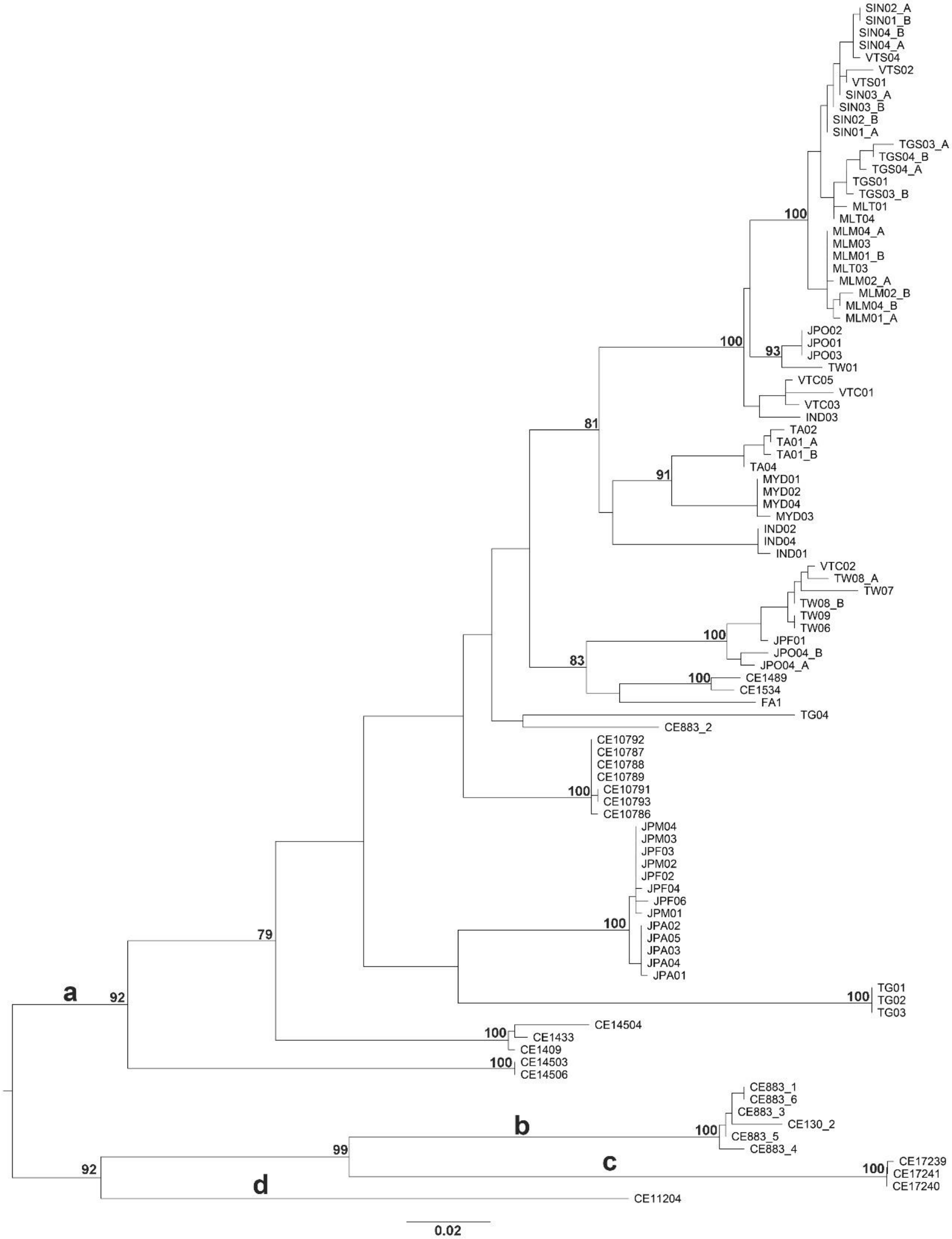

Supplementary Figure 1. A ML phylogenetic tree of *P. litoralis* based on the ITS2. The nodes with ML bootstraps > 70% are considered well-supported. The numbering is the input MOTUs of the BPP analyses, the letters a - d are the four most conservative MOTUs suggested by BPP.
